# Testing a model of benefit-finding and growth in youths with chronic health conditions

**DOI:** 10.1186/s12887-023-04467-3

**Published:** 2024-01-05

**Authors:** Roman E. von Rezori, Harald Baumeister, Reinhard W. Holl, Kirsten Minden, Annabel S. Müller-Stierlin, Christina Reinauer, Svenja Temming, Petra Warschburger

**Affiliations:** 1 Department of Psychology, Counseling Psychology, University of Potsdam, Karl-Liebknecht-Str. 24-25, 14476 Potsdam, Germany; 2 Department of Clinical Psychology and Psychotherapy, Institute of Psychology and Education, Ulm University, Ulm, Germany; 3 Institute for Epidemiology and Medical Biometry, ZIBMT, Ulm University, Ulm, Germany; 4 Department of Pediatric Respiratory Medicine, Immunology and Critical Care Medicine, Charité University Medicine Berlin, Berlin, Germany; 5 Department of Psychiatry and Psychotherapy II, BKH Günzburg, Ulm University, Günzburg, Germany; 6 Department of General, Pediatrics, Neonatology and Pediatric Cardiology, Medical Faculty, University Hospital Düsseldorf, Heinrich-Heine-University, Düsseldorf, Germany

**Keywords:** Benefit-finding and growth, Adolescents, Chronic Illness, Resilience, Coping

## Abstract

**Background:**

The experience of benefit-finding and growth (BFG), defined as perceiving positive life changes resulting from adversity, is increasingly studied among youths with chronic health conditions (CCs). However, empirical evidence is scarce for explaining individual differences in BFG. The study aimed to test a model of BFG, including an interplay of personal and environmental factors and coping processes.

**Methods:**

A sample of *N* = 498 youths (12–21 years) recruited from three German patient registries for CCs (type 1 diabetes: *n* = 388, juvenile idiopathic arthritis: *n* = 82, cystic fibrosis: *n* = 28) completed a questionnaire including self-reported optimism, social support from parents and peers, coping strategies, and BFG. The model was created to reflect the theoretical assumptions of the Life Crisis and Personal Growth model and current empirical evidence. Structural equation modeling was conducted to evaluate the incremental explanatory power of optimism, peer group integration, parental support, acceptance, cognitive reappraisal, and seeking social support over and above sociodemographic and disease-related characteristics.

**Results:**

The model (CFI = 0.93; RMSEA = 0.04; SRMR = 0.05) explained 32% of the variance in BFG. Controlling for sociodemographic and disease-related characteristics, acceptance, cognitive reappraisal, and seeking social support were directly and positively linked to BFG. All tested coping strategies significantly mediated the association between optimism and BFG, whereas seeking social support significantly mediated the relation between peer group integration and BFG.

**Discussion:**

The study stresses the prominent role of emotion-focused coping strategies and peer group integration in enhancing BFG in youths with CCs.

**Trial registration:**

German Clinical Trials Register (DRKS), no. DRKS00025125. Registered on May 17, 2021.

**Supplementary Information:**

The online version contains supplementary material available at 10.1186/s12887-023-04467-3.

## Background

Chronic health conditions (CCs) are characterized by their chronicity, functional impairments, absence of a cure or disease progression, physical disability or pain, and the need for permanent health care [1]. There is consistent evidence that living with CCs, irrespective of the specific diagnosis is associated with a greater vulnerability to psychosocial problems during childhood and adolescence [2, 3]. Furthermore, these psychosocial problems may be a precursor to mental health problems in adulthood, such as anxiety and depression [4]. Shifting the perspective to conditions and processes that foster positive subjective well-being and resilience, a growing body of evidence suggests that individuals facing CCs also perceive benefit-finding and growth following their diagnosis [5, 6]. The concept of benefit-finding and growth (BFG) refers to individual differences in perceiving positive life changes resulting from adversity [6]. These changes involve perceptions of intrapersonal growth (e.g., feeling stronger and wiser), interpersonal growth (e.g., feeling closer to family and friends), and changes in life priorities and goals [7]. BFG is based on theories of psychosocial adaptation to stressful life experiences and emerges when individuals search for the meaning of these challenges [7, 8]. However, no empirically confirmed model currently explains individual differences in BFG. It remains largely unknown why some individuals perceive BFG in the face of CCs, whereas others report more global distress and less well-being [5]. Given youths’ vulnerability to stress-related diseases, a more holistic understanding of underlying positive pathways is urgently needed. In disease prevention, knowledge about different response patterns to CCs could be essential for identifying potential targets for psychosocial interventions to promote resilience in people needing continuous health care.

Adolescence offers an essential opportunity for investigating the sources of BFG, given the cognitive, social, and emotional changes that occur during this developmental stage. Early adolescence to emerging adulthood may represent the cradle of BFG as young adolescents begin to cope with stressors actively and internally [9] and to form future-oriented thoughts and concerns [10], which may involve efforts to deal with the long-term psychosocial implications of their CC. A recent systematic review revealed 38 studies supporting the presence of BFG in pediatric medical populations [11]. The authors identified several factors that were associated with higher levels of BFG. These factors include optimism, social support provided by family members and peers, and emotion-focused coping strategies. Emotion-focused coping strategies involve seeking to reduce or manage the emotional consequences of stressors [12]. More precisely, studies suggest that individuals who try to accept their condition, cognitively reappraise emotional situations, and express their illness experience to significant others are more likely to perceive BFG [13–15]. A closer look at the interplay of these factors and BFG is required to gain a deeper understanding of the potential pathways to BFG.

**Fig. 1:**
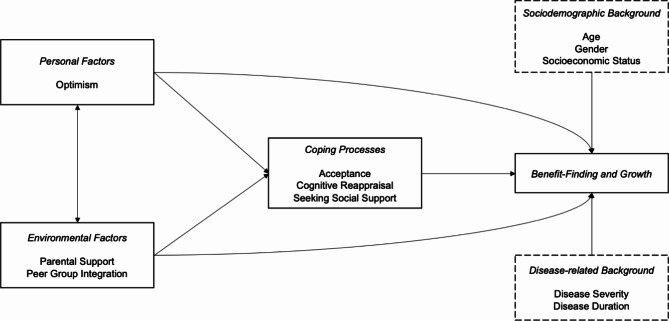
Adapted version of the “Life crisis and personal growth model” (Schaefer & Moos, 1992) that was tested in the present study

A theoretical framework describing this interplay is the “Life crisis and personal growth model” [16]. The model emphasizes a person-environment transaction and posits that personal factors, environmental factors, and coping processes directly explain the experience of BFG. Moreover, the model hypothesizes that personal and environmental factors indirectly influence BFG by affecting how individuals cope with their life crises. Therefore, the present study focuses on the direct and indirect pathways between optimism, social support provided by parents and peers, and the tendency to use acceptance, cognitive reappraisal, and seeking significant others when confronted with disease-related problems (see Fig. 1).

Despite the number of previous studies, several drawbacks need further empirical investigation. Most findings are based on bivariate correlations, and studies aiming to explain individual differences in BFG statistically are scarce [11]. Previous studies using multivariate regression models to explain BFG did not test a particular model of BFG and differed considerably in terms of constructs included in the model and the operationalization of these. In addition, these studies rarely included sociodemographic or disease-related control variables in their models. Although empirical evidence is mixed, age, gender, socioeconomic status, disease severity, and disease duration are discussed as critical correlates of BFG [11]. Controlling for these sociodemographic and disease-related characteristics would provide more robust support for an incremental contribution of optimism, social support, and coping strategies to explaining BFG. Furthermore, most studies examining BFG in youths with CCs were based on small sample sizes (ranging from *n* = 31 to *n* = 243), which may have led to unreliable results and invalid conclusions. To the best of our knowledge, no study so far has examined the complex interplay of empirically relevant personal and environmental factors and coping responses within one model explaining BFG. Although evidence from optimism and social support research highlights the mediating role of so-called approach coping responses, e.g., acceptance, cognitive reappraisal, or support seeking [17, 18], current research approaches do not conceptualize these as mediators.

Therefore, the present study aimed to test a structural model based on an adapted version of the “Life crisis and personal growth model” in a sample of youths diagnosed with CCs receiving routine care in clinical institutions across Germany. In contrast to previous studies, which mainly focused on cancer populations, we follow a non-categorical approach [19]. By including CCs with diverse illness characteristics, one can identify generic characteristics in the process of BFG. Especially the identification of generic modifiable aspects will facilitate the development of theory-driven, non-categorical interventions, which is crucial for adolescents with a rare condition.

## Methods

### Participants and procedure

This study was part of a consortium (trial registration: DRKS00025125). For further details, please refer to the study protocol [20]. Data were collected between June 2019 and November 2021 through an online questionnaire. Following data safety regulations, participants were recruited during their regular check-up visits in clinical centers, which are part of German patient registries for type 1 diabetes (T1D), juvenile idiopathic arthritis (JIA), or cystic fibrosis (CF). Inclusion criteria were as follows: participants’ age between 12 and 21 years, informed consent, and a medical diagnosis of T1D, JIA, or CF confirmed by a physician. Participants who were willing to complete the psychosocial assessment received an invitation email with a link to the online survey. Participants received gift coupons (20 Euros) as incentives. The study was approved by the University of Potsdam Ethics Committee.

### Measures

#### Benefit-finding and growth

BFG in response to CCs was assessed with the German translation of the Benefit Finding Scale for Children (BFSC; [21, 22]). The scale consists of 10 items (e.g., “Having had my illness has helped me learn to deal better with my problems.”). Responses were recorded on a 5-point Likert scale ranging from “not at all true for me” to “very true for me.” The internal consistency (McDonald’s ω) was ω = 0.90.

#### Optimism

Optimism was assessed with the eponymous scale of the Questionnaire of Resources in Childhood and Youth (FRKJ 8–16; [23]). The scale consists of six items (e.g., “I look to my future with confidence.”) rated on a 4-point Likert scale ranging from “never true” to “always true.” The internal consistency was ω = 0.90.

#### Social support

The quality of social contact with peers and the quality of social support by parents were measured by two subscales of FRKJ 8–16 [23]. Both scales (“peer group integration” and “parental support”) consist of six items each (e.g., “My friends like me the way I am.”; “When I need support, my parents are there for me.”) rated on a 4-point Likert scale ranging from “never true” to “always true.” The internal consistencies were as follows: ω = 0.87 (peer group integration), ω = 0.93 (parental support).

#### Coping strategies

*Acceptance* was assessed with the German version of the Coping with a Disease Inventory [24]. The acceptance scale consists of 6 items (e.g., “I accept my illness.”). The internal consistency in the present study was ω = 0.90. Participants rate their use of the coping strategies on a 5-point Likert scale ranging from “never “to “always.” The habitual use of *reappraisal* was assessed with the German version of the Emotion Regulation Questionnaire (ERQ; [25]) including six items (e.g., “When I want to feel more positive emotions, I change the way thinking I’m thinking about the situation.”). Items are scored on a 7-point Likert scale ranging from “strongly disagree” to “strongly agree.” The internal consistency reached ω = .80. The Berlin Social Support Scales [26] were used to assess *seeking social support* (five items, e.g., “When I am worried, I reach out to someone to talk to.”) on a 4-point Likert scale ranging from “strongly agree” to “strongly disagree.” The internal consistency was ω = .84.

#### Sociodemographic and disease-related data

Participants reported their sociodemographic and disease-related data. Perceptions of social status were measured with an adolescent version of the MacArthur Scale [27], which asks individuals to rank their familial placement within a 10-point society ladder ranging from “least money, little or no education, no job or jobs that no one wants or respects” to “most money, the highest amount of schooling, and the jobs that bring the most respect.” Subjective disease severity and age at diagnosis were assessed with single items (“I perceive my illness as severe.”/”How old were you when a doctor diagnosed your illness?“). Disease severity was rated on a 5-point Likert scale ranging from “not at all true for me” to “very true for me.”

#### Impact of the COVID-19 pandemic

Given that recruitment coincided with the COVID-19 pandemic, we matched survey data with the data on the stringency of COVID-19 containment measures in Germany extracted from the Oxford COVID-19 Government Response Tracker (OxCGRT) [28]. The stringency index records the strictness of lockdown policies ranging from 0 (no measures) to 100 (total lockdown).

### Data analysis

#### Preliminary analyses

Group differences in BFG were examined using one-way ANOVAs. Pearson’s bivariate correlation was used to examine the associations between BFG and sociodemographic and disease-related variables. Latent correlations were performed to analyze the relationship between BFG and optimism, coping variables, and social support variables. To test for dependencies in the data (multistage-sampling/medical diagnosis), we computed an intraclass correlation coefficient (ICC) by setting up a random intercept-only model for BFG [29].

#### Main analyses

Structural equation modeling (SEM) was carried out to test model. Therefore, we examined the direct and indirect effects of optimism, peer group integration, and parental support while controlling for age/disease duration, gender, social status, and disease severity. As age is confounded with disease duration (*r* = .29, *p* < .01), we tested an alternative model with disease duration instead of age as a predictor. Mediation is established if the bootstrap confidence interval of the indirect effect *a* × *b* does not include the zero [30].

All statistical analyses were performed using R (Version 4.1.0; R Core Team, 2021). SEM was conducted using the R package lavaan (Rosseel, 2012). We used maximum likelihood (ML) estimation and bootstrapping with 10.000 samples to estimate standard errors and 95% bias-corrected confidence intervals (CI) for parameter estimates of all models. Bootstrapping offers standard errors and non-symmetric confidence intervals, which are robust to nonnormality, yielding more accurate inferences and statistical power [31]. Because the χ^2^ test is sensitive to sample sizes, three indices were used to assess the model fit. Comparative fit index (CFI) of ≥ 0.90, root mean square error of approximation (RMSEA), and standardized root mean square residual (SRMR) of ≤ 0.08 were considered acceptable [32]. Allowing model identification, we fixed the path from the first indicator variable to the latent variable to 1. As the missing rate for each item was ≤ 1%, we conducted full information maximum likelihood analyses to account for the missing data. Overall, this method is preferable to conventional methods, yielding unbiased and efficient estimates [33].

## Results

### Sample characteristics

The final sample consisted of *N* = 498 participants aged 12 to 21 years (*M* = 15.43, *SD* = 2.07; *n* = 290 female (58.2%); *n* = 207 male (41.6%); *n* = 1 non-binary (0.2%)). Participants had a mean subjective social status of 6.61 (*SD* = 1.42; range = 1–10). Most participants were diagnosed with T1D (77.9%, *n* = 388), 16.5% (*n* = 82) were diagnosed with JIA, and 5.6% (*n* = 28) were diagnosed with CF.

**Table 1:** Descriptive statistics and correlations between BFG and all measured manifest variables

	*N*	*M (SD)*	Range		Correlations with BFG		
BFG	498	3.01 (0.96)	1–5		-		
Age	498	15.43 (2.07)	12–21		0.00		
Gender^1^	497	0.58 (0.49)	0–1		0.07		
Social status	498	6.61 (1.42)	1–10		0.16**		
Disease severity	498	2.54 (0.94)	1–5		− 0.04		
Disease duration^2^	498	7.55 (4.45)	0–20		− 0.06		
SI_OxCGRT	498	51.96 (24.08)	1-100		− 0.07		

*Note*. BFG = benefit finding and growth; CI = confidence interval; SI_OxCGRT = oxford COVID-19 government response tracker; T1D = type 1 diabetes; JIA = juvenile idiopathic arthritis; CF = cystic fibrosis; * *p* < .05. ** *p* < .01. ***p < .001; 1 = point-biserial correlation (0 = male, 1 = female); 2 = in years

### Preliminary analyses

There was no significant difference in level of BFG between participants who were surveyed before (*n* = 97; *M* = 3.14, *SD* = 0.96) and during the pandemic (*n* = 401; *M* = 2.98, *SD* = 0.95), *F*(1, 496) = 2.16, *p* = 14 (*d* = -0.17, 95% *CI*, -0.39 to 0.05) (cut-off date: 11th of March 2020 according to the WHO declaration). Descriptive statistics and manifest correlations between BFG and sociodemographic and disease-related data are presented in Table 1. See Table 2 for all latent correlations between all variables. The random intercept model with both clinical center/medical diagnosis as a grouping variable, demonstrated an ICC of less than 0.01, confirming the independence of residuals and absence of hierarchical data structure.

**Table 2:** Latent correlations between BFG and optimism, coping strategies, and social support by parents and peers

	*M* (*SD*)	Range	(1)	(2)	(3)	(4)	(5)	(6)
(1) BFG	3.01 (0.96)	1–5						
(2) Optimism	2.87 (0.72)	1–4	0.41***					
(3) Acceptance	4.02 (0.84)	1–5	0.33***	0.60***				
(4) Cognitive reappraisal	4.25 (1.11)	1–7	0.42***	0.53***	0.36***			
(5) Seeking social support	2.70 (0.72)	1–4	0.46***	0.58***	0.31***	0.47***		
(6) Peer group integration	3.35 (0.59)	1–4	0.24***	0.57***	0.38***	0.28***	0.55***	
(7) Parental support	3.43 (0.68)	1–4	0.31***	0.58***	0.29***	0.38***	0.49***	0.32***

*Note*. BFG = benefit-finding and growth; * *p* < .05. ** *p* < .01. ***p < .001

### Main analyses

#### Structural path model

**Fig. 2:**
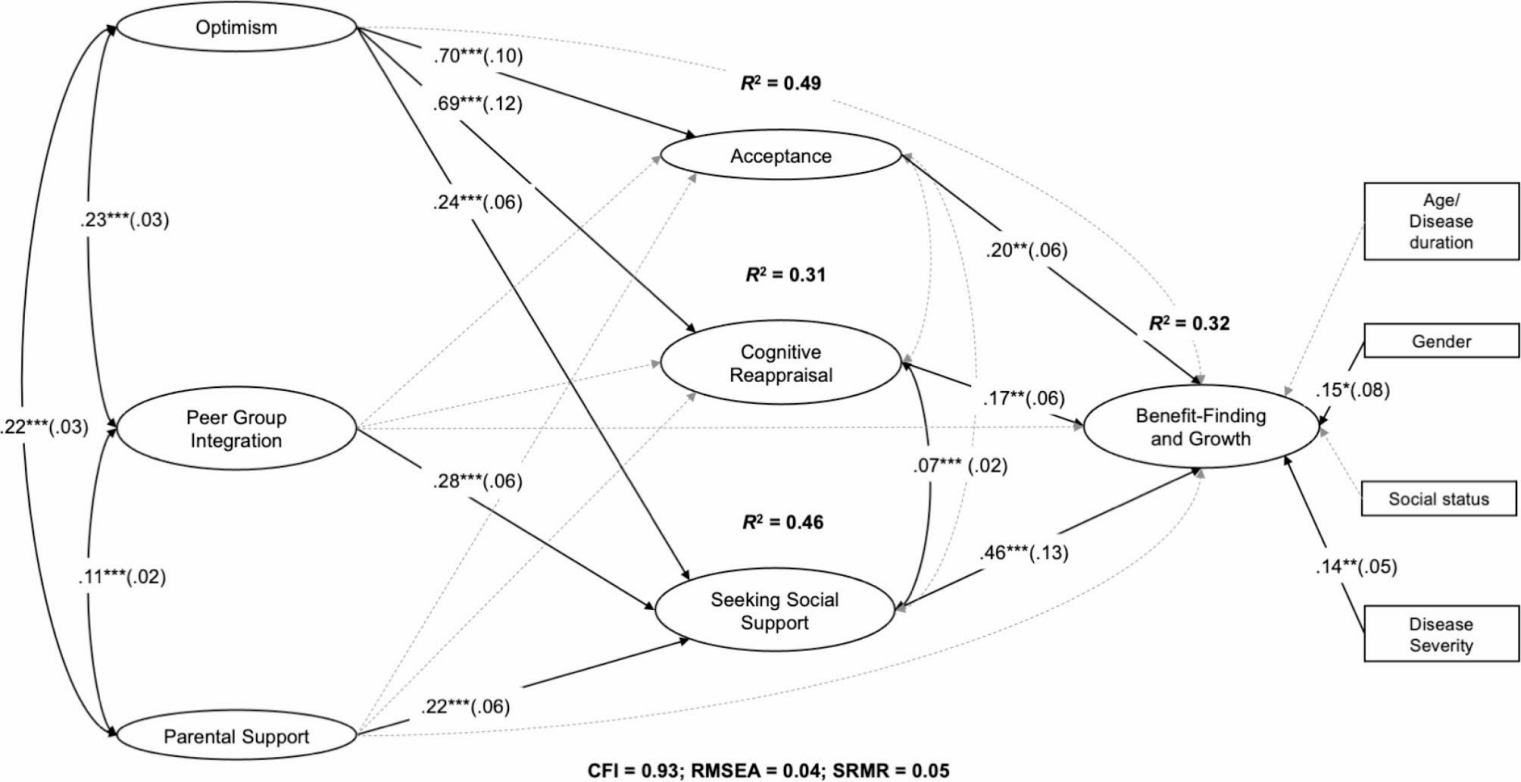
Structural paths model of benefit-finding and growth. For simplification, observed indicators of latent variables, errors, and thresholds were excluded from the figure; dashed lines = non-significant paths; numbers next to the arrows = standardized path coefficients, * *p* < .05. ** *p* < .01. ***p < .001, standard errors in parentheses; *R*^2^ next latent variables = explained variance; CFI = comparative fit index; RMSEA = root mean square error of approximation; SRMR = standardized root mean square error. Gender: male = 0, female = 1

The estimation of the structural path model of BFG (see Fig. 2) yielded an adequate model fit (CFI = 0.93; RMSEA = 0.04; SRMR = 0.05). In total, the model explained 32% of the variance in BFG, 49% of the variance in acceptance, 31% in reappraisal, and 46% in seeking social support. Detailed results are provided in the Supplementary Material (see A for results for a-paths, b-paths, direct effects, and correlations; see B for partial/total indirect effects, total effects, and contrasts; see C for results for control variables). Acceptance, cognitive reappraisal, seeking social support, gender (0 = male; 1 = female), and subjective disease severity had significant direct effects on BFG while controlling for other predictor variables (results did not significantly change with disease duration instead of age as predictor: CFI = 0.93; RMSEA = 0.04; SRMR = 0.05). Furthermore, significant total effects of optimism (c = 0.50, 95% CI [0.29, 0.73], *p* < .001) and peer group integration (c = 0.28, 95% CI [0.10, 0.46], *p* < .001) were observed. In comparison to peer group integration and parental support, optimism had a significantly higher total effect on BFG. In addition, optimism, peer group integration, and parental support were significantly and positively interrelated. After entering the mediators into the model, optimism predicted all mediators significantly, while peer group integration and parental support only predicted support seeking significantly. All mediators, in turn, predicted BFG significantly while controlling for age/disease duration, gender, social status, and subjective disease severity. The relationship between optimism and BFG was fully mediated by acceptance, reappraisal, and support seeking (total indirect effect *ab* = 0.37, 95% CI [0.24, 0.53], *p* < .001). Furthermore, the relationship between peer group integration and BFG was fully mediated by seeking social support (total indirect effect *ab* = 0.14, 95% CI [0.03, 0.25], *p* < .05). While the total effect of parental support was not significant, seeking social support partially mediated the link between parental support and BFG (*ab* = 0.10, 95% CI [0.04, 0.18], *p* < .01). In total, there were no significant differences between the effects of the mediators. Cognitive reappraisal was significantly and positively correlated with seeking social support.

## Discussion

The purpose of this study was to understand the differential effects of theoretically and empirically derived personal (optimism), environmental factors (social support), and coping responses (acceptance, cognitive reappraisal, and seeking social support) on individual differences in BFG among youths with CCs. Therefore, we tested a model of BFG, hypothesizing direct and indirect pathways to BFG.

### Direct pathways

We provide solid evidence suggesting that acceptance, cognitive reappraisal, and seeking social support are directly linked to BFG in youths with CCs, over and above sociodemographic and disease-related characteristics. Our results indicate that youths more likely to respond to disease-related stressors by such coping responses perceive higher levels of BFG. This further underlines the prominent role of emotion-focused coping strategies in adapting to the emotional consequences of uncontrollable or unchangeable stressors in the context of CCs [34].

### Indirect pathways

Additionally, our results supported the hypothesis that optimism and social support are indirectly associated with BFG. The analysis of indirect paths pointed towards differential effects. Optimism was positively associated with a tendency towards accepting the CCs, cognitively reappraising emotional situations, and expressing illness-related problems to significant others. This finding aligns with meta-analytic evidence from adult populations [35], suggesting that optimists may adjust their coping responses to approach the demands of stressors or emotions. Conversely, the pattern of results showed that youths who perceived higher levels of parental support and felt more socially integrated with their peer group reported a higher tendency to seek support when confronted with illness-related problems. Although the total effect was substantially higher for optimism compared to peer group integration and parental support, these factors were significantly interrelated. This pattern of results follows evidence revealing that optimists have more significant relationships and greater social networks compared to pessimists [17]. Moreover, previous work emphasizes that supporting others might help people to maintain a positive self-concept during serious illness by validating their experiences and shaping expectancies about future outcomes related to health [18].

### Sociodemographic and disease-related context

Although a certain developmental level and cognitive skills might be necessary to reflect on personal experiences and integrate positive elements into world views [6], our results indicate that the experience of BFG is independent of the adolescent’s age. Indeed, mixed results have been found across previous studies regarding age [11]. Likewise, we found no association between social status and BFG, despite theories suggest that BFG is an important personal strength for individuals with lower social status [36]. Based on previous research on BFG, disease duration is of great conceptual interest [6]. It is commonly assumed that individuals who have had more time to process the meanings and implications of their illness are more likely to perceive BFG. However, our results contradict this conceptual assumption. In contrast, female gender and higher levels of perceived disease severity were positively associated with BFG when other model factors were considered simultaneously. This is in line with previous meta-analytic evidence from adults showing that female gender and both objective (e.g., physician rating) and subjective disease severity (e.g., patient’s report) are positively associated with BFG [5].

### Strengths and limitations

The present study has several strengths. To the best of our knowledge, our study provides the first empirically tested model of BFG. The model attributes significant importance to person-environment interaction and coping processes in the context of CCs. Because researchers and clinicians are most notably interested in the ways youths cope with their CCs, indicator variables were used based on well-established self-report measures. Furthermore, our study included a broad age range and youths with three different medical diagnoses, enhancing the generalizability of our results. It should be further stressed that our relatively large sample size and methodically sound approach may yield more accurate inferences and statistical power than previous studies.

However, limitations must also be acknowledged. Our results are based on cross-sectional data, potentially mispresenting temporal processes. Therefore, hypothesized causal relations must be treated with caution. Longitudinal data are needed to confirm the proposed pathways in the model further. Nevertheless, recent evidence supports our interpretation by showing that social support and the use of approach coping styles significantly predicted BFG in adults with cancer [37]. Possibly limiting the generalizability of our results, our analyses precluded the role of the ethnic background of patients. US-American studies suggest that BFG might be more adaptive for people who are of minority ethnicity or race [5]. Summarily, we cannot rule out the importance of other coping behaviors due to, for example, cultural differences (e.g., religious or spiritual coping). Finally, some data were collected during the COVID-19 pandemic, which may have influenced the results of our study. There is growing evidence that the pandemic had an inverse effect on the mental health condition of children, adolescents, and adults [38]. Specific CCs (e.g., diabetes) were identified and communicated as a risk factor for severe complications from COVID-19 at an early stage during the pandemic, and children and adolescents with CCs were especially vulnerable to the mental health effects of the COVID-19 pandemic [39]. However, we found no difference in BFG levels between participants surveyed before vs. during the pandemic nor an association between BFG and strictness of lockdown measures.

### Implications for Clinical Practice

Providing an empirically tested model of BFG is relevant from a clinical perspective. The premise of growth from adversity is at the core of most therapeutic frameworks. In addition to the psychosocial problems of youths facing CCs, pediatric psychologists are also interested in highlighting, amplifying, and learning from their patients’ strengths. BFG represents such a strength, involving cognitive, emotional, and social skills that allow people to hold both negative and positive aspects of CCs. Promoting BFG is congruent with the goal of most evidence-based and already existing interventions. By focusing on the interplay of potentially modifiable personal and social resources and approach coping strategies, we present evidence to facilitate the development of targeted interventions to improve BFG in youths with CCs. To date, interventions aiming to enhance BFG are mainly delivered to parents of children with CCs [40] or only incorporate cognitive reappraisal [41]. Including elements of acceptance and commitment, social skills training, and peer support might improve the efficacy of future interventions.

## Conclusion

To sum up, we confirmed an adapted version of the “Life crisis and personal growth model” by empirical data of youths with T1D, JIA, and CF. Peer support, seeking social support, acceptance, and cognitive reappraisal may be a particularly worthwhile focus for interventions promoting BFG in youths with CCs. Further research utilizing longitudinal data is needed to deepen our understanding of the mechanisms behind BFG.

### Electronic supplementary material

Below is the link to the electronic supplementary material.

Supplementary Material 1

## Data Availability

Fully anonymized data will be available from the corresponding author on reasonable request and with the permission of the collaboration partners.
