# Supplementary material for: Testing a model of benefit-finding and growth in youths with chronic health conditions
Source: BMC Pediatr. 2024 Jan 5;24:19. doi: 10.1186/s12887-023-04467-3 (PMC10768283; doi:10.1186/s12887-023-04467-3)
Supplement: Supplementary file 1 — Supplementary Material 1 [file 12887_2023_4467_MOESM1_ESM.docx]

**SUPPLEMENTARY MATERIAL**

1. **Structural path analysis: results for a-paths, b-paths, direct effects, and correlations.**

|  |  |  | 95% confidence interval | |
| --- | --- | --- | --- | --- |
| Path | standardized parameter estimate | SE | lower | upper |
| Acceptance (a-paths) | | | | |
| Optimism | .70*** | .10 | .50 | .90 |
| Peer group integration | .11 | .09 | -.08 | .28 |
| Parental support | .13 | .10 | -.07 | .32 |
| Reappraisal (a-paths) | | | | |
| Optimism | .69*** | .12 | .47 | .94 |
| Peer group integration | .08 | .10 | -.10 | .28 |
| Parental support | .18 | .11 | -.02 | .40 |
| Support seeking (a-paths) | | | | |
| Optimism | .24*** | .06 | .13 | .37 |
| Peer group integration | .28*** | .06 | .16 | .40 |
| Parental support | .22*** | .06 | .11 | .33 |
| Benefit-finding and growth (b-paths) | | | | |
| Acceptance | .20** | .06 | .09 | .33 |
| Reappraisal | .17** | .06 | .05 | .30 |
| Support seeking | .46*** | .13 | .22 | .72 |
| Direct effects | | | | |
| Optimism | .13 | .12 | -.12 | .37 |
| Peer group integration | .14 | .09 | -.03 | .32 |
| Parental support | .05 | .09 | -.14 | .22 |
| Correlations | | | | |
| OPT & PGI | .23*** | .03 | .18 | .29 |
| PGI & PSU | .11*** | .02 | .07 | .16 |
| PSU & OPT | .22*** | .03 | .17 | .27 |
| ACT & ER | .06 | .03 | -.01 | .12 |
| ER & SUS | .07** | .02 | .03 | .11 |
| SUS & ACT | .00 | .02 | -.04 | .03 |

*Note*. * *p* < .05. ** *p* < .01. ****p* < .001. SE = standard error. OPT = optimism; ACT = acceptance; ER = reappraisal; SUS = support seeking; PGI = peer group integration; PSU = parental support.

1. **Structural path analysis: results for partial/total indirect effects, total effects, and contrasts.**

|  |  |  | 95% confidence interval | |
| --- | --- | --- | --- | --- |
|  | standardized parameter estimate | SE | lower | upper |
| Partial indirect effects | | | | |
| OPT × ACT | .14** | .05 | .06 | .25 |
| PIG × ACT | .02 | .02 | -.01 | .07 |
| PSU × ACT | .03 | .02 | -.08 | .01 |
| OPT × ER | .12* | .05 | .04 | .23 |
| PIG × ER | .01 | .01 | -.01 | .02 |
| PSU × ER | .03 | .02 | -.01 | .09 |
| OPT × SUS | .11** | .04 | .04 | .20 |
| PIG × SUS | .13** | .04 | .05 | .22 |
| PSU × SUS | .10** | .04 | .04 | .18 |
| Total indirect effects | | | | |
| OPT indirect | .37*** | .08 | .24 | .53 |
| PGI indirect | .14* | .06 | .03 | .25 |
| PSU indirect | .11 | .05 | .01 | .22 |
| Total effects c | | | | |
| OPT total | .50*** | .11 | .29 | .73 |
| PGI total | .28*** | .09 | .10 | .46 |
| PSU total | .16 | .09 | -.03 | .32 |
| Contrasts | | | | |
| OPT vs. PGI (total) | .24** | .10 | .07 | .44 |
| OPT vs. PSU (total) | .27** | .10 | .10 | .48 |
| PGI vs. PSU (total) | .03 | .07 | -.10 | .16 |
| ACT vs. ER (b-paths) | .03 | .09 | -.15 | .21 |
| ACT vs. SUS (b-paths) | -.26 | .14 | -.54 | .01 |
| ER vs. SUS (b-paths) | -.29 | .15 | -.60 | .02 |

*Note*. OPT = optimism; ACT = acceptance; ER = reappraisal; SUS = support seeking; PGI = peer group integration; PSU = parental support; Partial indirect effect = product of a- and b-paths; total indirect effect = sum of partial indirect effects of one antecedent; total effect c = sum of the direct and indirect effect; contrasts = subtraction of total indirect effects/b-paths; * *p* < .05. ** *p* < .01. ****p* < .001. SE = standard error.

1. **Structural path analysis: results for control variables**

|  |  |  | 95% confidence interval | |
| --- | --- | --- | --- | --- |
|  | standardized parameter estimate | SE | lower | upper |
| Benefit-finding and growth | | | | |
| Age (Time since diagnosis) | .00 (-.02) | .01 (.02) | -.02 (-.06) | .02 (.02)) |
| Gender | .15* | .08 | .01 | .30 |
| Social status | .04 | .03 | -.02 | .09 |
| Disease severity | .14** | .05 | .04 | .24 |
| Acceptance | | | | |
| Age (Time since diagnosis) | .01 (.10*) | .02 (.04) | -.03 (.01) | .05 (.18) |
| Gender | -.10 | .07 | -.24 | .04 |
| Social status | -.03 | .03 | -.09 | .02 |
| Disease severity | -.37*** | .04 | -.45 | -.28 |
| Reappraisal | | | | |
| Age (Time since diagnosis) | -.03 (-.02) | .02 (.04) | -.01 (-.11) | .08 (.06) |
| Gender | .16 | .08 | -.01 | .32 |
| Social status | .02 | .03 | -.05 | .08 |
| Disease severity | .05 | .05 | -.05 | .15 |
| Support seeking | | | | |
| Age (Time since diagnosis) | .15** (-.03) | .05 (.04) | .06 (-.10) | .24 (.04) |
| Gender | .01 | .02 | -.03 | .04 |
| Social status | .02 | .02 | -.01 | .05 |
| Disease severity | .09* | .04 | .02 | .16 |

*Note*. OPT = optimism; ACT = acceptance; ER = reappraisal; SUS = support seeking; PGI = peer group integration; PSU = parental support; Partial indirect effect = product of a- and b-paths; total indirect effect = sum of partial indirect effects of one antecedent; total effect c = sum of the direct and indirect effect; contrasts = subtraction of total indirect effects/b-paths; * *p* < .05. ** *p* < .01. ****p* < .001. SE = standard error.

1. **Acknowledgement for clinical centers**

**National Diabetes Registry**

- AKK Altonaer Kinderkrankenhaus (Pädiatrie, Diabetologie und Endokrinologie)
- Asklepios Kinderklinik Sankt Augustin (Allgemeine Kinder- und Jugendmedizin)
- Bad Mergentheim (Praxis für Kinder mit Diabetes Typ 1 und ihren Familien)
- Bethlehem Gesundheitszentrum Stolberg (Klinik für Kinder- und Jugendmedizin)
- Charité Universitätsmedizin Berlin (Campus Virchow-Klinikum, Sozialpädiatrisches Zentrum)
- Bürgerhospital Frankfurt am Main (Clementine Kinderhospital)
- Bürstadt (Praxis Dr. Kee)
- Katholisches Kinderkrankenhaus Wilhelmstift (Pädiatrie)
- Kinderarztpraxis Dr. Dörte Hilgard Witten
- Kinderklinik Garmisch-Patenkirchen (Sozialpädiatrisches Zentrum, Diabetesambulanz)
- Klinik Hallerwiese-Cnopfsche Kinderklinik Nürnberg
- Klinikum am Steinenberg Reutlingen (Klinik für Kinder- und Jugendmedizin)
- Kliniken Böblingen (Klinik für Kinder- und Jugendmedizin)
- Klinikum Chemnitz (Kinderendokrinologie und Kinderdiabetologie)
- Technische Universität Dresden (Klinik und Poliklinik für Kinder- und Jugendmedizin)
- Klinikum Konstanz (Klinik für Kinder und Jugendliche, Diabetes-Zentrum für Kinder und Jugendliche Region Bodensee)
- Lukaskrankenhaus Neuss
- Medicover Oldenburg Medizinisches Versorgungszentrum
- Medizinisches Versorgungszentrum Leopoldina Schweinfurt (Kinderdiabetologie)
- Universitätsklinikum Aachen (Klinik für Kinder- und Jugendmedizin)
- Universitätsklinikum Augsburg (Klinik für Kinder und Jugendliche)
- Universitätsklinikum Bonn (Zentrum für Kinderheilkunde)
- Universitätsklinikum Düsseldorf (Klinik für allgemeine Pädiatrie, Neonatologie und Kinderkardiologie)
- Universitätsklinikum Freiburg (Zentrum für Kinder- und Jugendmedizin, Endokrinologie)
- Universitätsmedizin Göttingen (Klinik für Kinder- und Jugendmedizin, Abteilung Neuropädiatrie)
- Universitätsklinikum Heidelberg (Zentrum für Kinder- und Jugendmedizin, Diabetesambulanz)
- Universitätsklinikum Schleswig-Holstein (Campus Kiel, Kinder- und Jugendmedizin I, Pädiatrische Endokrinologie und Diabetologie)

**National Paediatric Rheumatologic Database**

- Charité Universitätsmedizin Berlin (Campus Virchow-Klinikum, Otto-Heubner-Centrum für Kinder- und Jugendmedizin, Sozialpädiatrisches Zentrum - Rheumaambulanz)
- St. Josef-Stift (Abteilung für Kinder- und Jugendrheumatologie)
- Technische Universität Dresden (Klinik und Poliklinik für Kinder- und Jugendmedizin)
- Universitätsklinikum Tübingen (Klinik für Kinder- und Jugendmedizin, Rheumatologische Ambulanz)

**Cystic Fibrosis Registry**

- Charité Universitätsmedizin Berlin (Mukoviszidose Ambulanz – Christiane Herzog Zentrum Berlin)
- Medizinische Hochschule Hannover (Mukoviszidose Ambulanz)
- Universitätsklinikum Jena (Klinik für Kinder- und Jugendmedizin)
